# Kinetics of Mushroom Tyrosinase and Melanogenesis Inhibition by *N*-Acetyl-pentapeptides

**DOI:** 10.1155/2014/409783

**Published:** 2014-07-22

**Authors:** Ching-Yi Lien, Ching-Yu Chen, Shih-Ting Lai, Chin-Feng Chan

**Affiliations:** ^1^Department of Chemistry, National Chiayi University, Chiayi 60004, Taiwan; ^2^Department of Applied Cosmetology, Hungkuang University, No. 1018, Sector 6, Taiwan Boulevard, Shalu District, Taichung 43302, Taiwan

## Abstract

We investigated the kinetics of 4*N*-acetyl-pentapeptides, Ac-P1, Ac-P2, Ac-P3, and Ac-P4, regarding inhibition of mushroom tyrosinase activity. The peptides sequences of Ac-P1, Ac-P2, Ac-P3, and Ac-P4 were Ac-RSRFK, Ac-KSRFR, Ac-KSSFR, and Ac-RSRFS, respectively. The 4*N*-acetyl-pentapeptides were able to reduce the oxidation of l-DOPA by tyrosinase in a dose-dependent manner. Of the 4*N*-acetyl-pentapeptides, only Ac-P4 exhibited lag time (80 s) at a concentration of 0.5 mg/mL. The tyrosinase inhibitory effects of Ac-P4 (IC_50_ 0.29 mg/mL) were more effective than those of Ac-P1, Ac-P2, and Ac-P3, in which IC_50_s were 0.75 mg/mL, 0.78 mg/mL, and 0.81 mg/mL, respectively. Kinetic analysis demonstrated that all 4*N*-acetyl-pentapeptides were mixed-type tyrosinase inhibitors. Furthermore, 0.1 mg/mL of Ac-P4 exhibited significant melanogenesis inhibition on B16F10 melanoma cells and was more effective than kojic acid. The melanogenesis inhibition of Ac-P4 was dose-dependent and did not induce any cytotoxicity on B16F10 melanoma cells.

## 1. Introduction

Hyperpigmentation is a common melanogenesis disorder caused by excess melanin production by the enzyme tyrosinase [1, 2]. It causes browning in fruits and vegetables and can cause freckles, senior spots, and melasma in human cutaneous tissues [3, 4]. Tyrosinase (polyphenol oxidase, EC 1.14.18.10) is an enzyme that is widely distributed in microorganisms, animals, and plants [5]. Tyrosinase comprises 2 copper ions, each coordinately bonded to a distinct set of 3 histidine residues within the active sites [6, 7]. It can catalyze 2 reactions through hydroxylation of monophenol to* o*-diphenol and by oxidation of* o*-diphenol into the corresponding* o*-quinone [8].

Many tyrosinase inhibitors, such as dihydroxybenzene (HQ), kojic acid, and arbutin, have been applied in treatment of hyperpigmentation [9–13]. HQ is one of the most common depigmenting agents for melasma treatment and exhibits clinical efficacy [14]. However, HQ has also been observed to generate reactive oxygen species and is considered to be cytotoxic to melanocytes and potentially mutagenic to mammalian cells, causing skin irritation [15–17]. The other tyrosinase inhibitors, kojic acid and arbutin, have failed to exhibit efficacy in vivo because of poor skin penetration or have potential for causing contact dermatitis and erythema after long-term use [16, 18].

In recent years, natural amino acids and short-sequence peptides have been considered as attractive potential therapeutic candidates for the treatment of skin disorders [19]. Several amino acids, such as phenylalanine (Phe), have been reported to reduce melanin formation through competitive inhibition of tyrosinase or by reducing tyrosinase uptake [20, 21]. The peptides have manifold potential through the combination of short-chain amino acids [22, 23]. Previous studies have proved that proteins and peptides obtained from natural sources such as milk [24], honey [25, 26], and wheat are able to inhibit tyrosinase activity. They have also shown that several synthetic oligopeptides (constituting amino acids 3–10) exhibit competitive inhibition of tyrosinase [19]. However, structure-activity relationship between peptides and tyrosinase might differ dramatically, based on various peptide sequences, the number of amino acids, and C- or* N*-terminal modification of peptides [27, 28]. In previous reports, the* N*-acetyl group has provided a crucial structural component to analogs with no* N*-terminal blocking groups and lower neutrophil polarization activity [28]. The* N*-acetyl peptides obtained through either chemical synthesis or biosynthesis might greatly influence peptide solubility [29] and biological functions such as anti-inflammation and inhibition of myeloperoxidase [27, 28, 30].

In this study, we synthesized 4 pentapeptides, in which the* N*-terminal was modified using an acetyl group (Ac). The aim of this study was to investigate the kinetic inhibition of tyrosinase activity by 4*N*-acetyl-pentapeptides, which consisted of various sequences of serine (S), arginine (R), phenylalanine (F), and lysine (K). The melanogenesis inhibition in B16F10 melanoma cells also was examined.

## 2. Materials and Methods

### 2.1. Materials

L-3,4-dihydroxyphenylalanine (l-DOPA), mushroom tyrosinase (product number: T3824; the activity was ≥1000 Units/mg), kojic acid, dimethyl sulfoxide (DMSO), 3-(4,5-dimethylthiazol-2-yl)-2,5-diphenyltetrazolium bromide (MTT), alpha-melanocyte-stimulating hormone (*α*-MSH), phosphate buffered saline (PBS), Wang resin, triisopropylsilane (TIS), trifluoroacetic acid (TFA), and diethyl ether were purchased from Sigma-Aldrich Co. LLC. (St. Louis, MO, USA). Compounds 9-fluorenylmethyloxycarbonyl- (Fmoc-) protected amino acids, 2-(1H-benzotriazol-1-yl)-1,1,3,3-tetramethyluronium hexafluorophosphate (HBTU), and 1-hydroxybenzotriazole (HOBt) were purchased from the CPC Scientific, Inc. (Sunnyvale, CA, USA). The peptides, Ac-P1, Ac-P2, Ac-P3, and Ac-P4 (peptide sequences: Ac-RSRFK, Ac-KSRFR, Ac-KSSFR, and Ac-RSRFS, resp.), were kindly provided by Dr. Lien, Department of Applied Chemistry, National Chiayi University, Taiwan. B16F10 mouse melanoma cell lines were purchased from Bioresource Collection and Research Center (BCRC, Hsinchu, Taiwan).

### 2.2. Peptide Synthesis

The* N*-acetyl-pentapeptides (Table 1) were synthesized by NeoMPS (San Diego, CA, USA), using solid-phase (9H-fluoren-9-ylmethoxy)carbonyl chemistry [31]. Peptides were prepared through solid phase peptide synthesis (SPPS) with Fmoc chemistry, using an ABI 433A peptide synthesizer (Applied Biosystems, Foster City, CA, USA). Each peptide synthesis was automated and amino acids were attached sequentially to form the C- to the* N*-terminal ends on the active sites of Wang resin. For each amino acid attached, the Fmoc protection group was cleaved in the presence of piperidine. The carboxyl group of the next amino acid was activated by the HBTU/HOBt and then coupled to the amino group of the previous amino acid [31–33]. After synthesis, 10 mL of cleavage reagent containing 95% TFA, 2.5% H_2_O, and 2.5% TIS was added to release the peptide from the resin. After stirring for 1.5 h at room temperature, the mixture was filtered to collect the filtrate. Chilled diethyl ether (50 mL) was poured into the filtrate to precipitate the peptide. The peptide was collected through centrifugation and then washed thoroughly with the cold ether. The synthesized peptide was purified using a C-18 cartridge and freeze-dried before use.

### 2.3. Inhibitory Effect of* N*-Acetyl-pentapeptides on Mushroom Tyrosinase

In a 96-well plate, 20 *μ*L of 0, 0.01, 0.05, 0.1, 0.2, 0.5, and 1 mg/mL of* N*-acetyl-pentapeptides was mixed with 160 *μ*L of 1 mM l-DOPA. After 20 *μ*L of 212.65 *μ*g/mL of mushroom tyrosinase (in 50 mM potassium phosphate buffer) was added, the solutions were incubated at 25°C for 30 min. All experiments were conducted in triplicate. The absorbance was measured at 475 nm by using an ELISA reader (TECAN, Austria). The tyrosinase inhibitory activity was calculated using the following equation: [(Δ_control_ − Δ_sample_)/Δ_control_] × 100%. The IC_50_ value was determined using regression of a constructing dose-response curve at which 50% target activity is lost.

### 2.4. Kinetic Analysis of Tyrosinase Inhibition

In a 96-well plate, the kinetic properties of* N*-acetyl-pentapeptides (20 *μ*L of 0, 0.1, and 0.2 mg/mL) in the inhibition of tyrosinase (20 *μ*L of 212.65 *μ*g/mL) were determined using various concentrations of l-DOPA (160 *μ*L of 0.25, 0.5, 1, 2, and 4 mM) as substrates. The reaction mixture was measured using an ELISA reader at 475 nm for 10 min. All experiments were conducted in triplicate. The inhibition mechanism was assessed using Lineweaver-Burk plots, and the inhibition constants *K*_*IS*_ and *K*_*I*_ were obtained from second plots of the apparent 1/*V*_max⁡_ and apparent *K*_*m*_/*V*_max⁡_ against the inhibitor concentration, respectively, as described by Liao et al. [34].

### 2.5. Cell Viability Assay

B16F10 cells were cultured in Dulbecco's modified Eagle's medium (DMEM; Gibco Life Technologies, Carlsbad, CA, USA), supplemented with 10% fetal bovine serum, 100 units/mL of penicillin G, 100 *μ*g/mL of streptomycin, and 0.25 *μ*g/mL of amphotericin, and then incubated at 37°C with 5% CO_2_. The viability of cells treated with* N*-acetyl-pentapeptides was determined using an MTT assay. Briefly, 3 × 10^4^ B16F10 cells were seeded and adhered in 96-well plates. After 24 h, DMEM was removed and 200 *μ*L of various concentrations of fresh DMEM and* N*-acetyl-pentapeptide solution were added, and the cells were incubated for 24 h. After incubation, the medium was removed, and 100 *μ*L of MTT in PBS solution (0.5 mg/mL) was added to each well, and the cells were incubated for 40 min at 37°C. Subsequently, the MTT solution was removed and 100 *μ*L of DMSO was added to each well, and the plate was gently shaken to dissolve the formazan crystals. The absorbance of each well was measured at 570 nm, using the ELISA reader. All determinations were performed in triplicate.

### 2.6. Assessment of Melanin Content

Cellular melanin content was determined as described previously [35]. Briefly, 1 × 10^5^ B16F10 cells were seeded in 24-well plates and cultured at 37°C and 5% CO_2_ for 48 h. Subsequently, the cells were treated with 100 nM *α*-MSH for 24 h, followed by various concentrations (0, 0.1, 0.5, and 1 mg/mL) of Ac-P4 or kojic acid for 24 h. The cells were washed twice with PBS and cell pellets were then dissolved in 100 *μ*L of 1 N NaOH in 10% DMSO, for 1 h at 80°C. The relative melanin content was determined by measuring the absorbance at 405 nm, using the ELISA reader (TECAN, Austria). The melanin content was calculated using the following equation: (Δ_sample_/Δ_control_) × 100%. All determinations were performed in triplicate.

### 2.7. Statistical Analysis

Statistical evaluation was performed by running one-way ANOVA followed by the Duncan multiple range tests and analysis of variance, using SigmaStatR (Version 3.11.0, Systat Software Inc., San Jose, CA, USA). Statistical significance was determined by *P* < 0.05.

## 3. Results and Discussion

### 3.1. Inhibitory Effect of* N*-Acetyl-pentapeptides on Mushroom Tyrosinase

**Figure 1:**
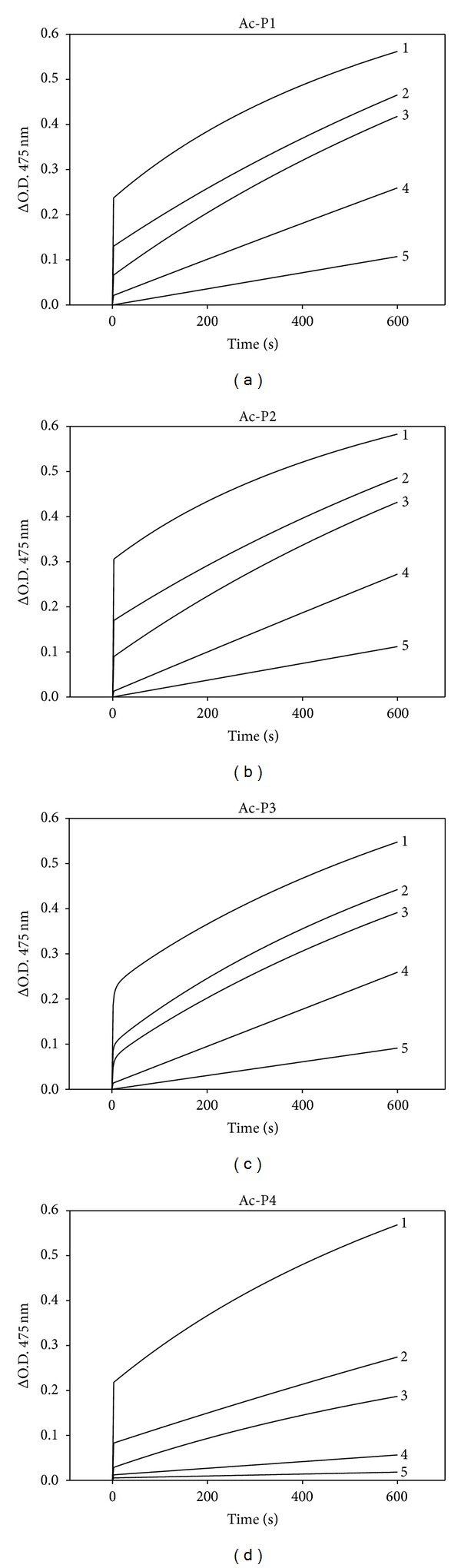
Inhibitory effects of the* N*-acetyl-pentapeptides on mushroom tyrosinase. (a) to (d) are progress curves of Ac-P1 to Ac-P4 for the oxidation of l-DOPA by the tyrosinase. The concentrations of inhibitor for curves 1–5 were 0, 0.1, 0.2, 0.5, and 1 mg/mL, respectively.

All 4*N*-acetyl-pentapeptides demonstrated potent inhibitory effects on mushroom tyrosinase activity and the inhibition was dose-dependent (Figures 1(a) to 1(d)). The IC_50_ of Ac-P4 was 0.29 mg/mL, which was significantly more potent than those of Ac-P1, Ac-P2, and Ac-P3 (Table 1). Only one peptide sequence difference was observed between Ac-P1 (Ac-RSRFK) and Ac-P4 (Ac-RSRFS) (Table 1). The results indicated that the phenylalanine-serine sequence in the C-terminal might play a critical role in the inhibitory effect on tyrosinase activity. The distinct tertiary structures of pentapeptides also may involve in regulation of tyrosinase activity. Therefore, the results of this study are not fully consistent with those of previous reports, which have shown that proline-serine (PS) and valine-serine (VS) dipeptides do not inhibit tyrosinase activity [36]. However, the 2 dipeptides have exhibited potent inhibitory effects on tyrosinase and melanin synthesis in Mel-Ab cells [36].

### 3.2. Kinetic Analysis of* N*-Acetyl-pentapeptides regarding Inhibitory Effects on Tyrosinase

**Figure 2:**
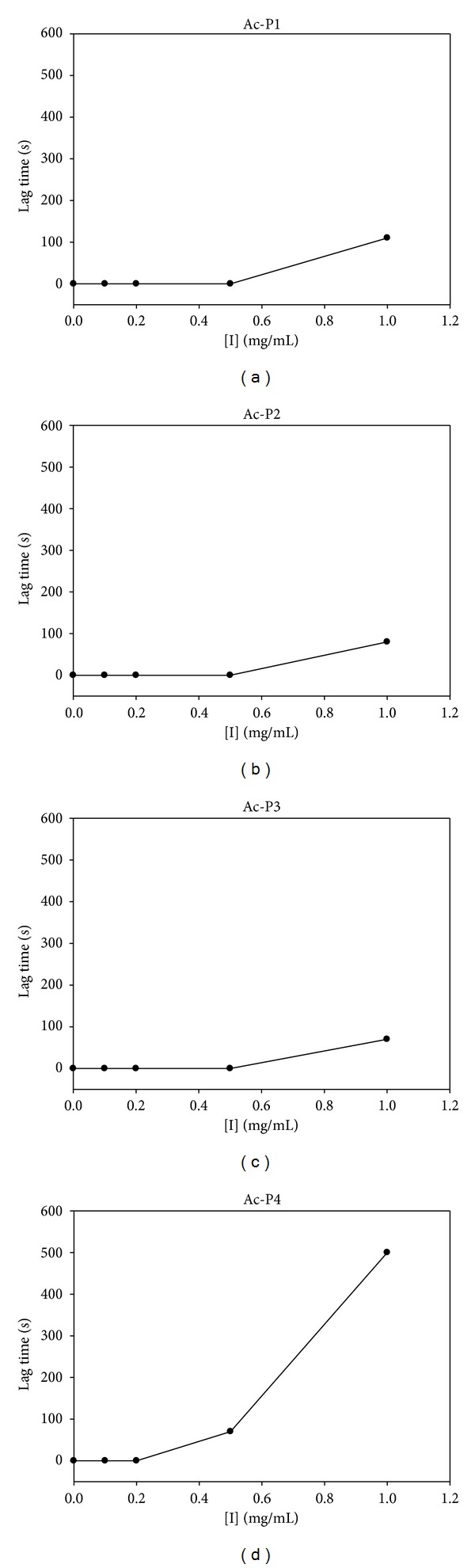
Effects of* N*-acetyl-pentapeptides on the lag time of mushroom tyrosinase. (a) to (d) are lag times of various concentration of Ac-P1 to Ac-P4.

No lag time was observed at the* N*-acetyl-peptide concentrations less than 0.5 mg/mL, except Ac-P4, for which the lag time was 80 s (Figure 2). Lag time is defined required to reach the steady state rate with respect to the diphenol concentration [8, 37]. The lag is influenced by substrate and enzyme concentration, enzyme source, pH of the medium, and presence of hydrogen donors, such as l-DOPA or other catechols, and transition metal ions [37, 38]. All 1 mg/mL (1.43–1.58 mM)* N*-acetyl-peptides exhibited lag time during enzyme reactions (Figure 2). The lag time of 1 mg/mL Ac-P4 was lengthened to 500 s and the lag times of Ac-P1, Ac-P2, and Ac-P3 were 110 s, 80 s, and 70 s, respectively (Figure 2). The Ac-pentapeptides may compete with l-DOPA for the activator site. However, the competition is not effective, except at millimolar concentrations or greater. The result indicated that l-DOPA is more specific for this site than Ac-pentapeptides, especially for Ac-P1, Ac-P2, and Ac-P3.

**Figure 3:**
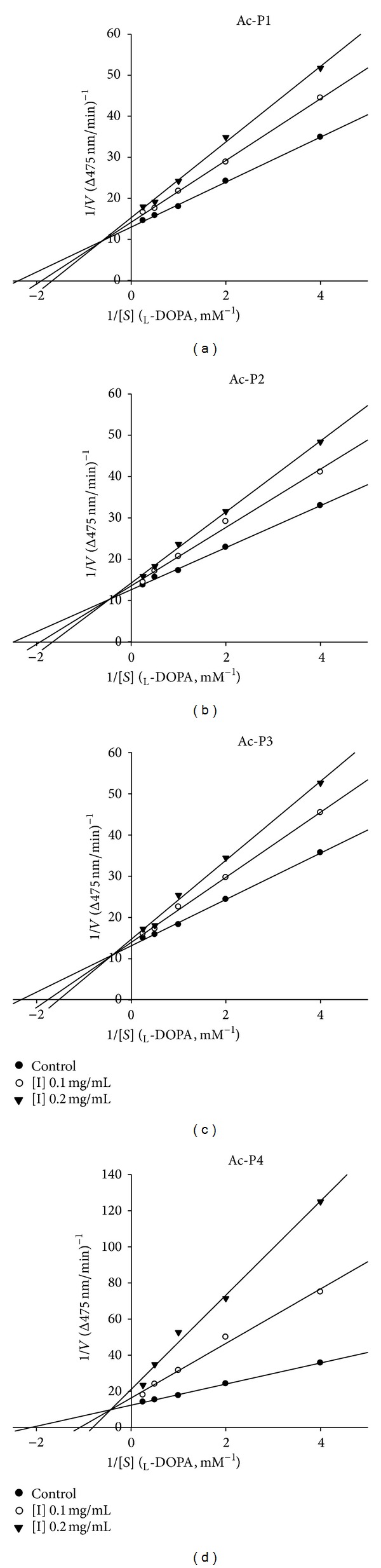
Lineweaver-Burk double reciprocal plot and determination of inhibition constants for Ac-P1 to Ac-P4 (a to d) on mushroom tyrosinase. The data include mean values of 1/*V*, and the inverse of the absorption increased at 475 nm per minute, in 3 independent tests with various concentrations of l-DOPA as a substrate. The reaction was observed in the presence of 0, 0.1, and 0.2 mg/mL of Ac-P1 to Ac-P4.

We adapted a Lineweaver-Burk plot analysis to elucidate inhibition types and mechanisms of Ac-P1 to Ac-P4 on tyrosinase. All the results showed changes in both the apparent *V*_max⁡_ and the *K*_*m*_, indicating that Ac-P1 to Ac-P4 induced a mixed type of inhibition (Figures 3(a) to 3(d)). The results indicated that the 4 peptides can bind to free enzymes and can also bind to a site distinct from the substrate of an enzyme-substrate complex. The secondary plots of slope and* Y*-intercept versus [*I*; (Ac-P1 to Ac-P4)] were linearly fitted. The *K*_*I*_ and *K*_*IS*_ of Ac-P1 to Ac-P4 were determined as 0.276, 1.278; 0.281, 2.003; 0.276, 1,685; and 0.053, 0.517 (Table 2). This result indicated that Ac-P4 might have stronger affinity to tyrosine or the tyrosine-l-DOPA complex than Ac-P1, Ac-P2, and Ac-P3 do.

Our results were not fully consistent with the results of previous reports in which short peptide inhibitors have been observed as competitive or noncompetitive inhibitors of tyrosinase [19, 25]. The kinetic difference might be due to the various peptide sequences and the* N*-acetyl blocking group of peptides, which might result in changing the affinity with tyrosinase. Previous reports have demonstrated that* N*-acetyl-peptides can serve as chelators for many divalent ions, such as copper ion [39]. Therefore, Ac-P4 might exhibit the same inhibition mechanism as that of kojic acid, which exhibits a mixed-type inhibitory effect on diphenolase [40, 41].

### 3.3. Cell Viability and Assessment of Melanin Content

**Figure 4:**
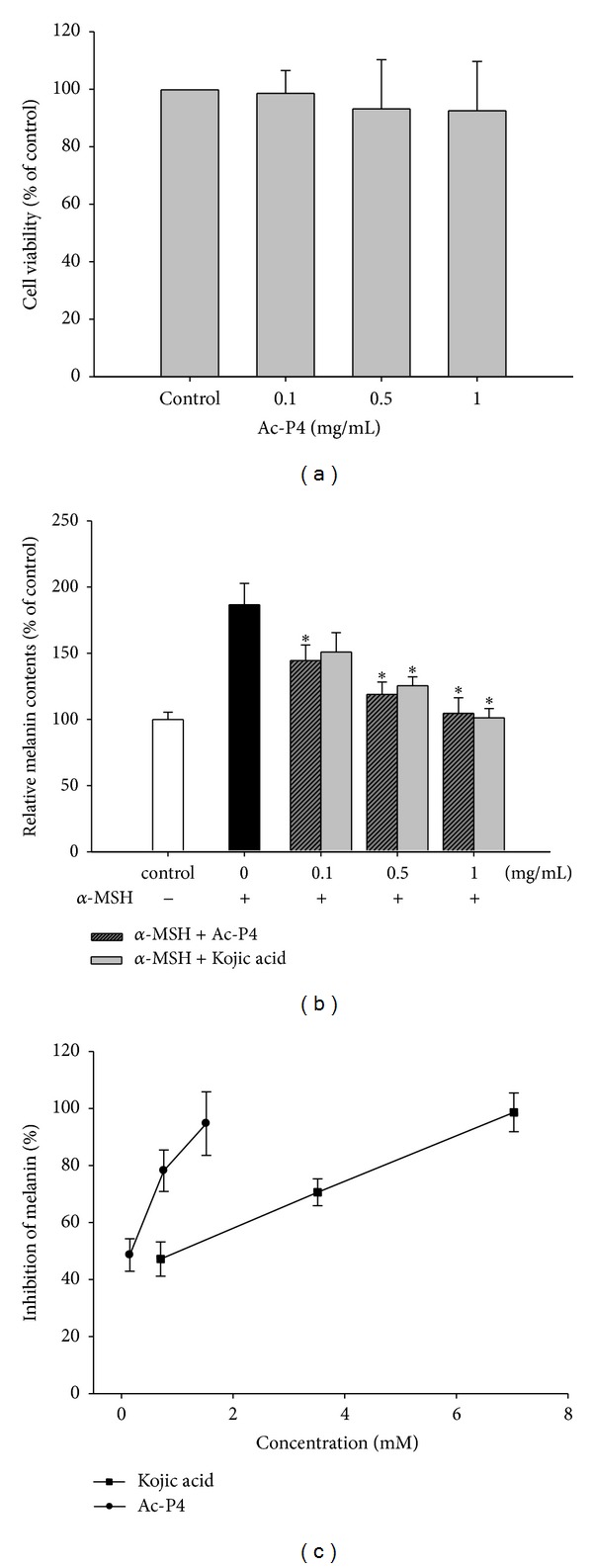
Effects of Ac-P4 on cell viability and melanin content of B16F10. (a) Effects of Ac-P4 on cell viability of B16F10 cells. B16F10 cells were treated with various concentrations of Ac-P4 for 24 h. Cell viability was determined using an MTT assay. Each percentage value in treated cells was calculated and compared with that in control cells. (b) Effects of Ac-P4 and kojic acid on cellular melanin content in B16F10 melanoma cells. The control readings were set at 100%. Data from experimental wells are expressed as percentages of the controls. Each column represents the mean ± SD of 3 independent experiments. *P < 0.05, compared with the *α*-MSH alone. (c) Reillustrated inhibitory effects of Ac-P4 and kojic acid on cellular melanin content of B16F10 in molarity (mM).

It has been reported that numerous tyrosinase inhibitors exhibit no inhibitory effects on melanin content analysis in melanoma cells. Therefore, Ac-P4 was further subjected to melanin content and a cell viability assay used to evaluate its inhibitory effect on melanogenesis. As shown in Figure 4(a), Ac-P4 did not induce any significant cytotoxicity in B16F10 cells at concentrations ranging from 0.1 to 1 mg/mL. The inhibitory effect of Ac-P4 on *α*-MSH-mediated melanogenesis was determined by measuring the quantity of intracellular melanin in the presence of *α*-MSH, as shown in Figure 4(b). The Ac-P4 substantially reduced the *α*-MSH-induced cellular melanin contents in a dose-dependent manner, compared with the group treated with *α*-MSH alone. A 0.1 mg/mL Ac-P4 can significantly inhibit *α*-MSH-induced melanogenesis and was even more effective than kojic acid (positive control) (Figure 4(b)). The inhibitory effects of Ac-P4 (molecular weight of 659) at 0.1 mg/mL (0.15 mM), 0.5 mg/mL (0.76 mM), and 1 mg/mL (1.52 mM) on melanin production were 48.63%, 78.18%, and 94.7%, respectively. The inhibitory effects of kojic acid (molecular weight of 142.1) at 0.1 mg/mL (0.70 mM), 0.5 mg/mL (3.51 mM), and 1 mg/mL (7.03 mM) on melanin production were 47.20%, 70.60%, and 98.64%, respectively. To consider the numerical differences of molecular mass, the inhibitory effects of Ac-P4 and kojic acid were reillustrated in molarity (Figure 4(c)). The inhibitory effects on melanin content showed significant difference between Ac-P4 and kojic acid. The IC_50_ for Ac-P4 and kojic acid was 0.09 mM and 1.51 mM, respectively. The results indicated that Ac-P4 was 16.7-fold stronger than kojic acid in inhibiting melanin formation.* N*-terminal peptide end of Ac-P4 is uncharged, compared to standard synthetic peptides permeability of cells increases which may associate with the potent inhibitory effect of Ac-P4 on melanin formation. The activity of kojic acid to inhibit melanin synthesis in cultured human melanocytes was considerably attenuated [42]. However, in human clinical studies kojic acid exhibited synergistic effects with other skin-lightening agents, such as hydroquinone or glycolic acid [43]. A combination of kojic acid with Ac-P4 may also exhibit synergistic effects on skin lightening and will be further investigated in a future study.

This study suggests that* N*-acetyl-pentapeptides can not only inhibit mushroom tyrosinase activity but also exhibit potent melanogenesis inhibition on B16F10 cells. It has been demonstrated that numerous* N*-acetyl-peptides exhibit potent biological activity, such as being chemoattractants or exerting an antifibrosis effect by binding to surface receptors [35, 44]. It is unclear whether Ac-P4 can competitively bind to membrane receptors such as melanocortin 1 receptor (MCR-1) and thereby repress alpha-melanocyte stimulating hormone- (*α*-MSH-) induced melanogenesis. The other melanogenesis inhibition mechanism of Ac-P4 was through directly inhibiting intracellular tyrosinase activity (supplemental data, available online at http://dx.doi.org/10.1155/2014/409783).

It has been reported that aloesin and hesperidin can not only effectively inhibit tyrosinase activity but also inhibit melanin formation on B16F10 and on normal human melanocytes [45, 46]. Furthermore, aloesin and hesperidin can significantly improve skin hyperpigmentation in vivo in 15 days and in one week, respectively [45, 46]. In this study, Ac-P4 has exhibited the great potential on reducing hyperpigmentation. Further investigations on mechanism of melanogenesis inhibition of Ac-P4 both in vitro and in vivo should be conducted in the future.

## 4. Conclusion

In this study, 4*N*-acetyl-pentapeptides exhibited potent inhibitory effects on mushroom tyrosinase, and the inhibition kinetics occurred through the same mechanism. Furthermore, melanogenesis inhibitory assays were performed using B16F10 cells. The results indicated that Ac-P4 might serve as a new potent depigmentation agent in cosmetics or food industries.

## Supplementary Material

Ac-P4 inhibited *α*-MSH-induced intracellular tyrosinase activity in a dose-dependent manner. 1 mg/ml Ac-P4 significantly inhibited intracellular tyrosinase activity by 57.9% (P<0.05).

## Figures and Tables

**Table 1:** *N*-Acetyl-pentapeptides inhibitory profile against diphenolase activity of mushroom tyrosinase.

*N*-Acetyl-pentapeptide	Peptide sequence	IC_50_ (mg/mL)
Ac-P1	Ac-RSRFK	0.75 ± 0.16
Ac-P2	Ac-KSRFR	0.78 ± 0.14
Ac-P3	Ac-KSSFR	0.81 ± 0.02
Ac-P4	Ac-RSRFS	0.29 ± 0.03∗

Data are means ± SD of three replicates. Significantly different at ∗*P* < 0.05, compared to Ac-P1. Level as determined by one-way ANOVA followed by Duncan multiple range tests.

**Table 2:** The inhibition constants and inhibition type for *N*-acetyl-pentaptides on mushroom tyrosinase.

*N*-Acetyl-pentapeptide	*K*_*I*_ (mg/mL)	*K*_*IS*_ (mg/mL)	Inhibition type
Ac-P1	0.276	1.278	Mixed
Ac-P2	0.281	2.003	Mixed
Ac-P3	0.276	1.685	Mixed
Ac-P4	0.053	0.517	Mixed

## Abbreviation

Ac-P1 to Ac-P4: * N*-Acetyl-pentapeptides
IC_50_: Half maximal inhibitory concentration
HQ: Dihydroxybenzene
Phe: Phenylalanine
AC: Acetyl group
S: Serine
R: Arginine
F: Phenylalanine
K: Lysine
l-DOPA: L-3,4-Dihydroxyphenylalanine
DMSO: Dimethyl sulfoxide
MTT: 3-(4,5-Dimethylthiazol-2-yl)-2,5-diphenyltetrazolium bromide
*α*-MSH: Alpha-melanocyte-stimulating hormone
PBS: Phosphate buffer solution
TIS: Triisopropylsilane
TFA: Trifluoroacetic acid
Fmoc: 9-Fluorenylmethyloxycarbonyl- (Fmoc-) protected amino acids
HBTU: 2-(1H-Benzotriazol-1-yl)-1,1,3,3-tetramethyluronium hexafluorophosphate
HOBt: 1-Hydroxybenzotriazole
SPPS: Solid phase peptide synthesis
DMEM: Dulbecco's modified Eagle's medium
PS: Proline-serine
VS: Valine-serine
MCR-1: Melanocortin 1 receptor.
