# Supplementary material for: Kinetics of Mushroom Tyrosinase and Melanogenesis Inhibition by N-Acetyl-pentapeptides
Source: ScientificWorldJournal. 2014 Jul 22;2014:409783. doi: 10.1155/2014/409783 (PMC4130364; doi:10.1155/2014/409783)
Supplement: Supplementary file 1 — Ac-P4 inhibited α-MSH-induced intracellular tyrosinase activity in a dose-dependent manner. 1 mg/ml Ac-P4 significantly inhibited intracellular tyrosinase activity by 57.9% (P<0.05). [file 409783.f1.pdf]

Supplemental data- TSWJ/409783.v1

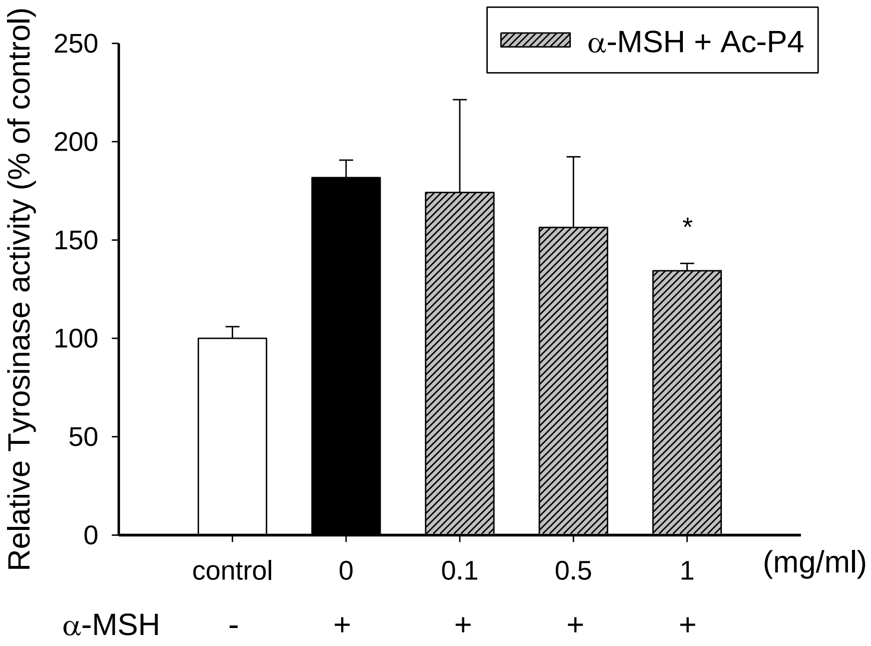

Fig 1. Inhibitory effects of Ac-P4 on α-MSH-induced tyrosinase activity in B16F10 cells. Ac-P4 inhibited α-MSH-induced tyrosinase activity in a dose-dependent manner and 1 mg/ml Ac-P4 significantly inhibited intracellular tyrosinase activity by 57.9% (P<0.05).

Experimental Procedure

Measurement of intracellular tyrosinase activity

The cells were seeded in 24-well plates (8 × 10^4 cells/well), and cultured in 37 °C, 5% CO_2 for 24 h. Then the cells were treated with 100 nM α-MSH for 24 h and then added Ac-P4 for 24 h. Cells rinsed with PBS twice and lysed with phosphate buffer (pH 6.8) containing 1% Triton X-100 followed by freezing and thawing three times. Centrifugation at 15,000 rpm for 10 min obtained clarified lysates. Cell lysate (90 μL) mixed with fresh prepared L-DOPA (10 μL) at 37 °C for 30 min. Tyrosinase activity was evaluated by the absorbance at 475 nm using an ELISA reader (Tecan, Austria).

Tyrosinase activity (%) = (△Abssample/△Abscontrol) ×100%.
